# Trends in Cardiac Arrest Mortality Among Young Adults in the United States, 1999 to 2024

**DOI:** 10.1016/j.jacadv.2026.102973

**Published:** 2026-07-03

**Authors:** Aimen Shafiq, Mushood Ahmed, Eeshal Zulfiqar, Maryam Shahzad, Tallal Mushtaq Hashmi, Vivek Bhat, Stephen J. Greene, Robert J. Mentz, Marat Fudim, Jamal S. Rana, Gregg C. Fonarow, Ankur Kalra

**Affiliations:** a Department of Medicine, Dow University of Health Sciences, Karachi, Pakistan; b Department of Medicine, Rawalpindi Medical University, Rawalpindi, Pakistan; c Department of Medicine, SUNY Upstate Medical University, Syracuse, New York, USA; d Division of Cardiology, Duke University Medical Center, Durham, North Carolina, USA; e Duke Clinical Research Institute, Durham, North Carolina, USA; f Division of Cardiology, Kaiser Permanente Northern California, Oakland, California, USA; g Division of Research, Kaiser Permanente Northern California, Oakland, California, USA; h Ahmanson-UCLA Cardiomyopathy Center, Division of Cardiology, University of California Los Angeles, Los Angeles, California, USA; i Division of Cardiology, Department of Medicine, SUNY Upstate Medical University, Syracuse, New York, USA

**Keywords:** cardiac arrest, CDC WONDER, mortality, United States

## Abstract

**Background:**

Cardiac arrest (CA) affecting young individuals has been identified as a public health concern in the United States.

**Objectives:**

This study analyzes mortality trends associated with CA in young adults in the United States, focusing on demographic and regional disparities.

**Methods:**

The Centers for Disease Control and Prevention Wide-Ranging Online Data for Epidemiologic Research database was used to extract death certificate data for individuals aged 15 to 44 years from 1999 to 2024. Age-adjusted mortality rates (AAMRs) were determined, and annual percentage changes were generated using JoinPoint regression analysis.

**Results:**

From 1999 to 2024, a total of 289,237 deaths were attributed to CA among young individuals in the United States. The AAMR remained stable from 1999 to 2018 but experienced a significant rise between 2018 to 2021, annual percentage change: 16.50% (95% CI: 8.88%-19.87%), followed by a decline through 2024. Males consistently exhibited higher AAMRs than females (11.06 vs 7.33). Among racial and ethnic groups, non-Hispanic (NH) Black or African American individuals had the highest average AAMR (18.68), followed by Hispanic/Latino populations (7.92), NH White individuals (7.81), and NH other populations (6.00). Geographically, the Western region had the highest AAMR (10.47) in 2024, whereas rural areas consistently recorded higher AAMRs than urban areas (12.04 vs 8.30).

**Conclusions:**

CA-related mortality in those aged 15 to 44 years exhibited fluctuating trends, with a notable rise between 2018 and 2021, followed by a decline through 2024. Males, NH Black Americans, and rural areas had higher mortality rates.

## Introduction

Cardiac arrest (CA) has been identified as a public health concern in the United States within the young population.^1^ Although CA is normally associated with increasing age and chronic cardiovascular conditions, recent reports suggest that CA is an important cause of death in younger individuals.^2^ Compared with older adults, where the burden of cardiovascular risk factors and atherosclerosis may drive risk of CA, for younger individuals, CA is often the first manifestation of an underlying cardiac abnormality, such as a familial cardiomyopathy or arrhythmia disorder. In addition, higher rates of substance abuse in younger populations may contribute to CA without pre-existing cardiovascular disease.^3^ These premature deaths pose indirect costs and consequences on society, with the loss of individuals at the peak of their physical and social life.^2^

Current literature has focused heavily on older populations, with fewer studies exploring trend or mortality data from CA in young adults.^4^ Existing data on trends in younger populations are heterogeneous, with some contemporary studies demonstrating stable or declining incidence in certain cohorts.^5^^,^^6^ The identification of temporal patterns and demographic disparities of deaths from CA can be useful in developing early detection strategies and public health interventions. Despite advances in preventive cardiology and emergency response systems, there is limited evidence to guide age-specific interventions for young adults.^7^

To address this, the Centers for Disease Control and Prevention Wide-Ranging Online Data for Epidemiologic Research (CDC WONDER) database was analyzed to examine the mortality trends associated with CA in young adults in the United States. The analysis stratifies findings by age, sex, race, and geographic location to elucidate the disparities. By identifying these trends, the study aims to guide clinical interventions and public health policies to improve awareness, prevention strategies, and patient outcomes in this population.

## Methods

### Study setting and population

Deaths occurring within the United States related to CA were extracted from the CDC WONDER database. CDC WONDER is an exhaustive repository of death certificate data from fifty states in the United States, as well as the District of Columbia. The Multiple Cause-of-Death Public Use Record death certificates were studied to select CA as a contributing or underlying cause of death on nationwide death certificates using International Statistical Classification of Diseases-10th Revision (ICD-10), Clinical Modification code I46 in younger adults aged 15 to 44 years at the time of death.^8^ These age groups have been used in prior studies to analyze mortality trends among young adults.^9^ Institutional Review Board approval was not required for this study, as we used a publicly available, deidentified data set provided by the government. Ethical approval for this study was not required. The study adheres to the reporting standards outlined in the Strengthening the Reporting of Observational Studies in Epidemiology (STROBE) guidelines.^10^

### Data abstraction

Data on CA-related deaths and population sizes were extracted. Supplemental Table 1 provides an overview of the variables available in death certificates. The inclusion criteria were defined as all deaths occurring in the United States between 1999 and 2024 among individuals aged 15 to 44 years in which CA (ICD-10-Clinical Modification code I46) was recorded as either an underlying or contributing cause of death on the death certificate. The exclusion criteria were deaths occurring outside the specified age range, deaths in which CA was not listed as an underlying or contributing cause, and records with missing or suppressed demographic or geographic data in accordance with CDC WONDER reporting standards. Demographics (sex, race/ethnicity, and age), and regional information (urban-rural and state) were extracted from 1999 to 2024. Race/ethnicities were delineated as non-Hispanic (NH) White, NH Black or African American, NH others (NH Asian or Pacific Islander, NH American Indian or Alaska Native, etc), and Hispanics or Latinos. These race/ethnicity categories have previously been used within analyses from the CDC WONDER database and rely on reported data on death certificates. Trends in mortality from CA were evaluated based on state-specific variations, U.S. census regions (Northeast, Midwest, South, and West), and county-level urbanization classifications. Counties were categorized as rural (micropolitan, noncore regions) or urban (large central metro, large fringe metro, medium metro, small metro) based on the 2013 National Center for Health Statistics Urban-Rural Classification Scheme.^11^ CA-related mortality in this study includes both out-of-hospital and in-hospital events, as the CDC WONDER database does not provide information to distinguish between these settings.

### Statistical analysis

Age-adjusted mortality rates (AAMRs) were calculated on an annual basis (per 100,000 individuals) by standardizing the CA-related deaths to the 2000 U.S. population as previously described.^12^ The Joinpoint Regression Program (Joinpoint, V 5.1.0.0; National Cancer Institute) was used to determine trends in AAMRs using annual percent change (APC). This method identifies significant changes in AAMRs over time by fitting log-linear regression models where temporal variation occurred. Joinpoint models were fitted using the standard software settings with a maximum of 0 to 4 joinpoints and permutation test-based model selection. APCs with 95% CIs for the AAMRs and crude mortality rates (CMRs) were calculated at the identified line segments linking join points using the Monte Carlo permutation test. APCs were considered increasing or decreasing if the slope describing the change in mortality was significantly different from zero using 2-tailed t-testing. Statistical significance was set at *P* < 0.05.

## Results

### Overall

**Figure 1:**
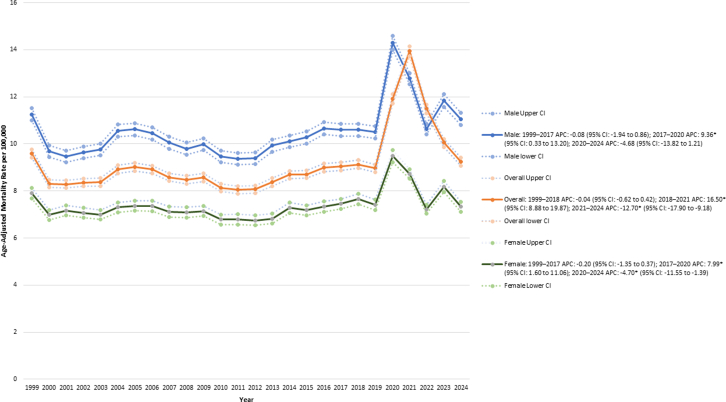
Overall and Sex-Stratified Cardiac Arrest-Related Age-Adjusted Mortality Rates per 100,000 Individuals in the United States, 1999 to 2024 ∗Data for urbanization AAMRs was unavailable for 2021-2024. APC = annual percent change.

From 1999 to 2024, a total of 289,237 CA-related deaths were reported among young adults in the United States (Supplemental Table 2). Overall, the AAMR remained stable from 1999 to 2018 (APC: −0.04% [95% CI: −0.62% to 0.42%]; *P* = 0.79), followed by a sharp increase from 9.13 in 2018 to 13.94 in 2021 (APC: 16.50% [95% CI: 8.88%-19.87%]; *P* < 0.001) (CMR: 8.52-13.21). Subsequently, the AAMR declined significantly to 9.24 in 2024 (APC: −12.70% [95% CI: −17.90% to −9.18%]; *P* < 0.001) (CMR: 13.21-8.75) (Supplemental Table 3, Figure 1). Regarding place of death, most CA-related deaths occurred in medical facilities (n = 217,875; 78.58%), followed by decedents’ homes (n = 46,674; 16.83%), nursing homes (n = 8,272; 2.98%), and hospice facilities (n = 3,179; 1.15%) (Supplemental Table 4).

### CA-related AAMR stratified by sex

Throughout the study period, males consistently had a higher average AAMR than females. In males, the AAMR remained stable from 1999 to 2017 (APC: −0.08% [95% CI: −1.94% to 0.86%]; *P* = 0.64), followed by a significant increase from 10.60 in 2017 to 14.29 in 2020 (APC: 9.36% [95% CI: 0.33-13.20]; *P* = 0.048) (CMR: 9.79-13.29), and then a nonsignificant decline to 11.06 through 2024 (APC: −4.68% [95% CI: −13.82-1.21]; *P* = 0.069) (CMR: 13.29-10.46).

A similar pattern was observed in females, with stable AAMRs from 1999 to 2017 (APC: −0.20% [95% CI: −1.35-0.37]; *P* = 0.38), followed by a significant increase from 7.45 in 2017 to 9.48 in 2020 (APC: 7.99% [95% CI: 1.60-11.06]; *P* = 0.024) (CMR: 6.95-8.98), and then a significant decline to 7.33 through 2024 (APC: −4.70% [95% CI: −11.55 to −1.39]; *P* = 0.033) (CMR: 8.98-6.99) (Supplemental Table 5).

### CA-related AAMRs stratified by race/ethnicity

Over the study period, NH Black or African-American individuals had the highest average CA-related AAMR (18.71), followed by Hispanic or Latino (7.92), NH White (7.81), and NH other populations (6.00).

Among NH Black or African-American individuals, the AAMR declined modestly from 22.39 in 1999 to 18.33 in 2018 (APC: −0.76% [95% CI: −1.84 to −0.04]; *P* = 0.041) (CMR: 21.17-16.17), followed by a significant increase to 26.85 in 2021 (APC: 16.87% [95% CI: 6.57-21.97]; *P* = 0.007) (CMR: 16.17-24.64), and then a subsequent decline to 18.18 through 2024 (APC: −12.47% [95% CI: −22.61 to −6.65]; *P* = 0.008) (CMR: 24.64-16.97).

The Hispanic or Latino group demonstrated a significant decline in AAMR from 9.43 in 1999 to 7.30 in 2018 (APC: −0.87% [95% CI: −1.73 to −0.11]; *P* = 0.024) (CMR: 8.09-6.66), followed by a sharp increase to 13.92 in 2021 (APC: 26.66% [95% CI: 16.23-32.03]; *P* < 0.001) (CMR: 6.66-12.70), and then a significant decline to 8.40 through 2024 (APC: −16.46% [95% CI: −22.54 to −11.62]; *P* < 0.001) (CMR: 12.70-7.59).

For NH White individuals, AAMR increased from 7.52 in 1999 to 8.08 in 2018 (APC: 0.49% [95% CI: 0.07-0.81]; *P* = 0.022) (CMR: 7.71-7.63), rose further to 11.59 in 2021 (APC: 12.82% [95% CI: 7.35-15.40]; *P* < 0.001) (CMR: 7.63-11.20), and then declined significantly to 7.88 through 2024 (APC: −11.20% [95% CI: −14.999 to −8.08]; *P* < 0.001) (CMR: 11.20-7.67).

**Figure 2:**
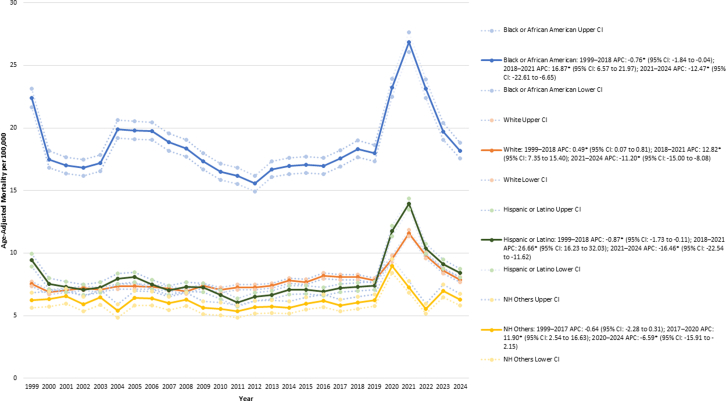
Cardiac Arrest-Related Age-Adjusted Mortality Rates per 100,000 Individuals Stratified by Race/Ethnicity in the United States, 1999 to 2024 ∗Data for urbanization AAMRs was unavailable for 2021-2024. NH = non-Hispanic; other abbreviation as in Figure 1.

In NH other populations, the AAMR remained stable from 1999 to 2017 (APC: −0.64% [95% CI, −2.28-0.31]; *P* = 0.14), increased significantly to 8.98 in 2020 (APC: 11.90% [95% CI, 2.54-16.63]; *P* = 0.024) (CMR: 5.87-8.87), and then declined significantly to 6.28 through 2024 (APC: −6.59% [95% CI, −15.91 to −2.15]; *P* = 0.030) (CMR: 8.87-6.35) (Supplemental Table 6, Figure 2).

### CA-related AAMRs stratified by geographical location

#### State-wide

From 1999 to 2020, states in the top 90th percentile for CA-related death AAMR included Mississippi, Nevada, New York, Georgia, and Alabama, whereas those in the bottom 10th percentile included Maryland, Wisconsin, Vermont, Oregon, and Minnesota. Between 2021 and 2022, Mississippi, Alabama, Arkansas, Nevada, and Georgia remained in the top 90th percentile, whereas Rhode Island, Utah, Vermont, the District of Columbia, and Maine ranked in the bottom 10th percentile. In 2023, the top 90th percentile states included Mississippi, Alabama, Nevada, Arkansas, and Alaska, whereas Rhode Island, Oklahoma, Utah, West Virginia, and Connecticut were in the bottom 10th percentile (Supplemental Table 7).

#### Census region

From 1999 to 2020, the Northeast had the highest AAMR, followed by the South, West, and Midwest. Between 2021 and 2022, the South recorded the highest AAMR, followed by the West, Northeast, and Midwest, whereas in 2023, the West had the highest rate, followed by the South, Northeast, and Midwest.

In the Northeast, AAMR declined significantly from 13.79 in 1999 to 8.68 in 2013 (APC: −2.47% [95% CI: −7.88 to −1.41]; *P* = 0.008) (CMR: 14.05-8.03), and subsequently remained stable through 2024. In the West, rates remained stable from 1999 to 2017 (APC: 0.35% [95% CI: −0.70-1.05]; *P* = 0.44), followed by a significant increase from 9.13 in 2017 to 12.80 in 2020 (APC: 9.79% [95% CI: 2.50-13.16]; *P* = 0.033) (CMR: 8.55-12.17), and then a nonsignificant decline through 2024 (APC: −2.93% [95% CI: −10.05-0.89]; *P* = 0.070) (CMR: 12.17-10.07).

**Figure 3:**
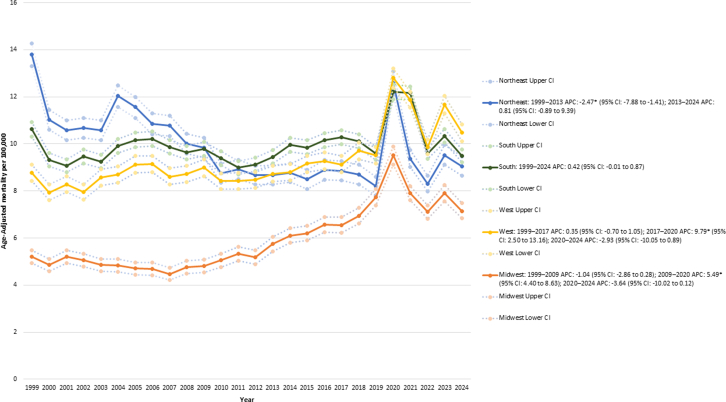
Cardiac Arrest-Related Age-Adjusted Mortality Rates per 100,000 Individuals Stratified by Census Region in the United States, 1999 to 2024 ∗Data for urbanization AAMRs was unavailable for 2021-2024. Abbreviation as in Figure 1.

In the South, the AAMR demonstrated a statistically nonsignificant increase over the study period (APC: 0.42% [95% CI: −0.006-0.87]; *P* = 0.051), ranging from 10.62 in 1999 to 9.50 in 2024 (CMR: 10.47-9.02). In the Midwest, AAMR remained stable from 1999 to 2009 (APC: −1.04% [95% CI: −2.86-0.28]; *P* = 0.12), followed by a significant increase from 4.82 in 2009 to 9.51 in 2020 (APC: 5.49% [95% CI: 4.40-8.63]; *P* < 0.001) (CMR: 4.52-8.87), and then remained stable through 2024 (APC: −3.64% [95% CI: −10.02-0.12]; *P* = 0.056) (CMR: 8.87-6.74) (Supplemental Table 8, Figure 3).

#### Urban-rural

From 1999 to 2020, rural areas consistently demonstrated higher AAMRs than urban areas. In urban regions, the AAMR remained stable from 1999 to 2018 (APC: −0.24% [95% CI: −1.29-0.31]; *P* = 0.31), before increasing significantly from 8.64 in 2018 to 11.11 in 2020 (APC: 16.06% [95% CI: 2.60-22.51]; *P* = 0.003) (CMR: 8.06-10.46).

**Figure 4:**
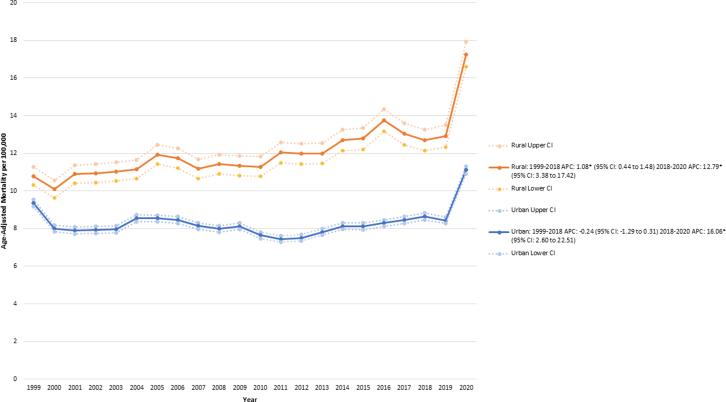
Cardiac Arrest-Related Age-Adjusted Mortality Rates per 100,000 Individuals Stratified by Urbanization in the United States, 1999 to 2020 ∗Data for urbanization AAMRs was unavailable for 2021-2024. Abbreviation as in Figure 1.

In rural areas, the AAMR increased significantly from 10.79 in 1999 to 12.70 in 2018 (APC: 1.08% [95% CI: 0.44-1.48]; *P* = 0.014) (CMR: 10.65-11.66), followed by a further marked increase to 17.25 in 2020 (APC: 12.79% [95% CI: 3.38-17.42]; *P* < 0.001) (CMR: 11.66-15.98). Urban-rural classification data from the CDC WONDER database were only available through 2020; therefore, urbanization-specific analyses could not be extended through 2024 (Supplemental Table 9, Figure 4).

## Discussion

This study presents CA-related mortality trends in the United States., focusing on individuals aged 15 to 44 years (Central Illustration). The observed AAMRs have fluctuated with a sharp increase from 2018 to 2021, followed by a significant decline through 2024. These trends warrant further exploration, as young adults represent an important population, and understanding the underlying causes is crucial for tailoring prevention strategies.

**Central Illustration:**
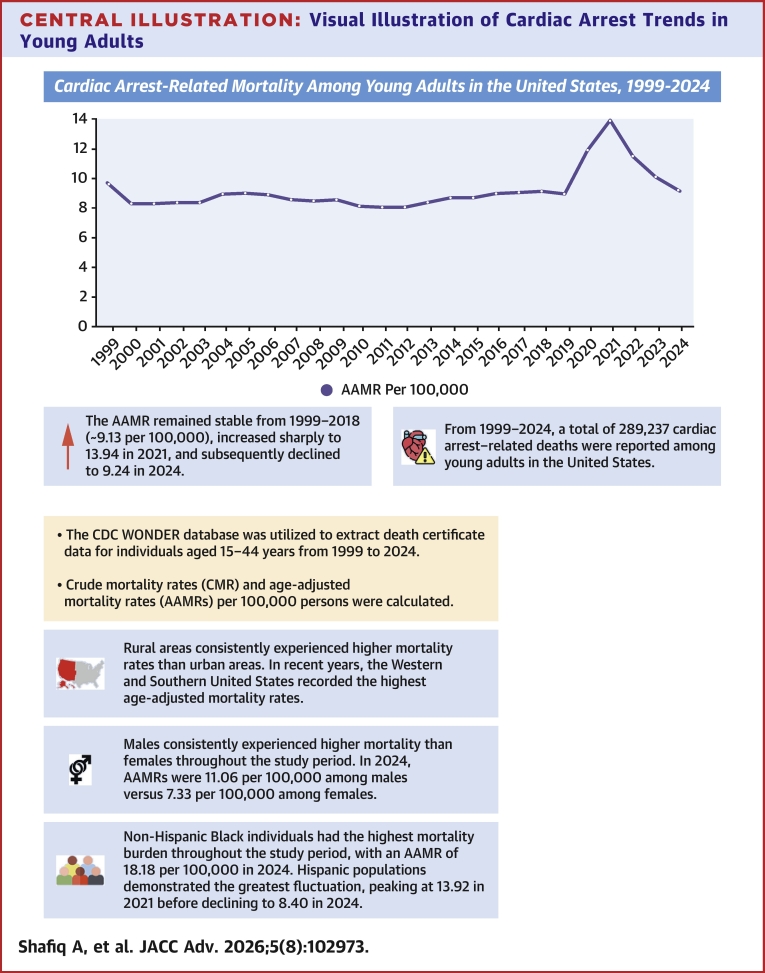
Visual Illustration of Cardiac Arrest Trends in Young Adults

Although CA mortality is often associated with elderly populations, the increasing trend in mortality among young adults is alarming. The higher CA-related mortality observed in recent years compared with 1999 may reflect a combination of long-term epidemiologic and systemic factors. Over the study period, there may have been a gradual increase in baseline cardiovascular vulnerability among younger populations, resulting in a larger pool of individuals at risk for fatal arrhythmic events. In parallel, improvements in diagnostic recognition and more precise attribution of cardiac causes of death on death certificates in recent years may have contributed to higher reported mortality in the later study period compared with earlier years. Collectively, these factors may partly explain the observed long-term increase in CA-related mortality between 1999 and 2023.

Traditional risk factors, such as coronary artery disease and hypertension, are less prevalent among the younger age groups compared with older populations; however, emerging evidence indicates that genetic arrhythmias, congenital heart abnormalities, and lifestyle-related causes such as obesity and sedentary lifestyles may be underlying causes of increased mortality from CA in this age group.^13^^,^^14^ The increase in obesity and its concomitant physical inactivity among adolescents and young adults not only directly relates to an increase in cardiovascular risk factors but also to hypertension and diabetes. It is well-recognized that obesity contributes to arrhythmias, which include atrial fibrillation and ventricular arrhythmias that can precipitate CA.^15^^,^^16^ In addition, abuse of substances, such as stimulants, including cocaine and amphetamines, is a major risk for the occurrence of CA in young persons.^17^ Increasing rates of e-cigarette and vaping use among the youth may be associated with a higher risk of acute coronary events, as evidence suggests these exposures can exacerbate cardiovascular disease and promote arrhythmias.^18^

Our analysis reaffirms the results of prior studies that suggest that men are generally at higher risk for CA than women.^19^ Traditionally, young men had much higher rates of CA, largely because they had higher rates of coronary artery disease, hypertension, and smoking.^20^

Racial, socioeconomic, and health care access disparities likely play a central role in the observed mortality patterns. Individuals from lower socioeconomic status experience higher rates of out-of-hospital CA and worse survival, driven by differences in baseline cardiovascular risk, access to preventive care, and emergency response systems.^21^ Structural inequities, including neighborhood-level deprivation, lower availability of automated external defibrillators, and reduced rates of bystander cardiopulmonary resuscitation,^21^ disproportionately affect Black and Hispanic populations and may contribute to the higher mortality observed in these groups. Disparities in access to advanced therapies, such as implantable cardioverter-defibrillators, further widen these gaps, leaving high-risk individuals less likely to receive life-saving interventions.^22^^,^^23^ These inequities may be particularly consequential in young adults, where delayed recognition of inherited or structural heart disease often reflects gaps in access to longitudinal care rather than biological differences alone.

Geographic patterns varied over time, with the Northeast showing the highest AAMRs earlier in the study period, followed by higher rates in the South and West during more recent years. This is likely influenced by a combination of lifestyle factors, health care disparities, and regional health policies. The Southern United States is known for having some of the highest rates of obesity,^24^ smoking, and physical inactivity, all of which increase the risk of CA. In addition, rural areas in these regions face significant health care access barriers, including fewer emergency medical services (EMS)^25^ and long transport times to hospitals, which can be critical when it comes to the survival of individuals experiencing CA. Expanding EMS networks and implementing community-based programs for early defibrillation could help mitigate these disparities.

The rising trend of CA mortality in young adults demands a reassessment of clinical practices, particularly with respect to early screening, prevention, and community-based interventions. In addition, expanding access to defibrillators in high-risk environments like schools, gyms, and public spaces is critical for improving survival rates. Lifestyle modifications, including increased physical activity, smoking cessation, and improved diet, should be prioritized as part of preventive care for young adults at risk for cardiovascular disease.

### Study limitations

Limitations of this study should be noted. The reliance on ICD codes and death certificates may have introduced misclassification of the cause of death, as these sources depend on accurate documentation by health care providers. Importantly, ICD-10 code I46 identifies CA as a cause of death but does not necessarily distinguish the underlying etiology. Because CA is often a terminal event rather than a primary diagnosis, some deaths coded under I46 may have reflected noncardiac or secondary causes, including drug overdose, trauma, asphyxia, pulmonary embolism, or other sudden deaths. This limitation may have influenced the observed temporal trends, particularly the sharp increase observed between 2018 and 2021. In addition, changes in coding practices, diagnostic recognition, health care utilization, and death certification patterns during the COVID-19 pandemic period may also have contributed to fluctuations in reported mortality rates. Therefore, the findings should be interpreted as trends in death certificate-reported CA mortality rather than definitively adjudicated arrhythmic or primary cardiac deaths. In addition, the database also lacks granular information on clinical characteristics, such as vital signs, laboratory results, imaging findings, or genetic testing, which could provide deeper insights into the underlying causes of CA. Moreover, the absence of data on social determinants of health limits the ability to evaluate their potential influence on health care access and disparities in outcomes among different racial and ethnic groups. The CDC WONDER database does not differentiate between out-of-hospital and in-hospital CA, precluding separate analysis of these distinct clinical entities, which may differ in underlying mechanisms, management, and outcomes. Moreover, sensitivity analyses restricted to underlying vs contributing causes of death were not feasible within the constraints of the CDC WONDER database and study design, which may limit the interpretability of cause-specific mortality attribution. Urban-rural classification data from the CDC WONDER database were only available through 2020; therefore, urbanization-specific analyses could not be extended through 2024, limiting direct comparison of these trends with other demographic and geographic analyses performed over the full study period. In addition, multiple stratified analyses were performed across demographic and geographic subgroups, increasing the possibility of type I error due to multiple comparisons. Therefore, subgroup findings should be interpreted cautiously and considered exploratory or hypothesis-generating rather than confirmatory. Lastly, AAMRs were standardized to the 2000 U.S. standard population, which facilitates comparability with prior studies and allows consistent evaluation of temporal trends over time. However, demographic shifts in the U.S. population over the study period, particularly population aging, may limit how well this reference population reflects the contemporary population structure and could therefore introduce some discrepancy in absolute rate estimates.

## Conclusion

Mortality due to CA among individuals aged 15 to 44 years showed variable trends, with a significant increase observed from 2018 to 2021, followed by a decline through 2024. Our study revealed that NH Black or African American young adults consistently experienced the highest CA-related AAMR over the study period. The Hispanic or Latino group showed sharp increases in mortality from 2018 to 2021, with a subsequent decline in 2024. Geographically, states such as Mississippi, Nevada, New York, Georgia, and Alabama had the highest mortality rates, whereas rural areas exhibited significantly higher rates compared with urban areas, particularly after 2018. These trends highlight the need for targeted prevention and intervention strategies, especially in high-risk communities and rural areas.

Perspectives **COMPETENCY IN MEDICAL KNOWLEDGE:** This study highlights that cardiac arrest in young adults, although often under-recognized, is a significant cause of premature mortality in the United States, with males, racial/ethnic minorities, and individuals in rural or high-mortality regions at particular risk. Clinicians should consider early identification of high-risk individuals through family history, genetic screening for arrhythmic syndromes or cardiomyopathies, and assessment of lifestyle risk factors. These findings emphasize the need for targeted preventive strategies, including community-based defibrillator programs and strengthened emergency medical services.

**TRANSLATIONAL OUTLOOK:** Translating these insights into practice faces challenges such as limited awareness of cardiac arrest risk in young adults, disparities in access to genetic testing, and variable availability of public defibrillators. Future research should focus on evaluating population-level screening strategies, improving bystander response and EMS coverage, and assessing the impact of lifestyle and preventive interventions to reduce disparities and improve survival outcomes in this vulnerable population.

## Funding support and author disclosures

Dr Fonarow reported receiving personal fees from 10.13039/100000046Abbott, 10.13039/100002429Amgen, 10.13039/100004325AstraZeneca, 10.13039/100004326Bayer, 10.13039/100001003Boehringer Ingelheim, 10.13039/100014941Cytokinetics, Eli Lilly, 10.13039/100004331Johnson & Johnson, 10.13039/100004374Medtronic, 10.13039/100004334Merck, 10.13039/100004336Novartis, and 10.13039/100004319Pfizer outside the submitted work. Dr Fudim has received personal fees from Alleviant, Ajax, Alio Health, Alleviant, Artha, Audicor, Axon Therapies, Bayer, Bodyguide, Bodyport, Boston Scientific, Broadview, Cadence, Cardioflow, Cardionomics, Coridea, CVRx, Daxor, Deerfield Catalyst, Edwards LifeSciences, Echosens, EKO, Feldschuh Foundation, Fire1, FutureCardia, Galvani, Gradient, Hatteras, HemodynamiQ, Impulse Dynamics, Intershunt, Medtronic, Merck, NIMedical, NovoNordisk, NucleusRx, NXT Biomedical, Orchestra, Pharmacosmos, PreHealth, Presidio, Procyreon, ReCor, Rockley, SCPharma, Shifamed, Splendo, Summacor, SyMap, Verily, Vironix, Viscardia, and Zoll; and has received grants from the National Institutes of Health, Doris Duke, outside the submitted work. Dr Mentz has received research support and honoraria from Abbott, American Regent, Amgen, AstraZeneca, Bayer, Boehringer Ingelheim, Boston Scientific, Cytokinetics, Fast BioMedical, Gilead, Innolife, Eli Lilly, Medtronic, Medable, Merck, Novartis, Novo Nordisk, Pfizer, Pharmacosmos, Relypsa, Respicardia, Roche, Rocket Pharmaceuticals, Sanofi, Verily, Vifor, Windtree Therapeutics, and Zoll. Dr Greene has received research support from the Duke University Department of Medicine Chair’s Research Award, American Heart Association, Amgen, AstraZeneca, Boehringer Ingelheim, Bristol Myers Squibb, Cytokinetics, Merck, Novartis, Otsuka, Pfizer, and Sanofi; has served on advisory boards or as consultant for Amgen, AstraZeneca, Bayer, Boehringer Ingelheim, Bristol Myers Squibb, Corcept, Corteria Pharmaceuticals, CSL Vifor, Cytokinetics, Eli Lilly, Lexicon, Merck, Novo Nordisk, Otsuka, Roche Diagnostics, Sanofi, scPharmaceuticals, Tricog Health, and Urovant Pharmaceuticals; and has received speaker fees from AstraZeneca, Bayer, Boehringer Ingelheim, Cytokinetics, Lexicon, and Roche Diagnostics. All other authors have reported that they have no relationships relevant to the contents of this paper to disclose.
