# Supplementary material for: Trends in Cardiac Arrest Mortality Among Young Adults in the United States, 1999 to 2024
Source: JACC Adv. 2026 Jul 3;5(8):102973. doi: 10.1016/j.jacadv.2026.102973 (PMC13355456; doi:10.1016/j.jacadv.2026.102973)
Supplement: Supplemental Tables 1-9 [file mmc1.pdf]

Supplementary appendix

Supplemental Table 1: Overview of Variables Available in Death Certificate Data

Supplemental Table 2: Cardiac arrest-related deaths, Stratified by Sex and Race in the United States, 1999 to 2024

Supplemental Table 3: Annual percent change (APC) of Cardiac arrest-related age-adjusted mortality rates per 100,000 in the United States, 1999 to 2024

Supplemental Table 4: Trends in Cardiac Arrest related mortality among young adults in the United States Stratified by Location of Death, 1999 to 2024

Supplemental Table 5: Overall and Sex-Stratified Cardiac arrest-related Age-Adjusted Mortality Rates per 100,000 in the United States, 1999 to 2024

Supplemental Table 6: Cardiac arrest- related Age-Adjusted Mortality Rates per 100,000, Stratified by Race in the United States, 1999 to 2024

Supplemental Table 7: Cardiac arrest-related Age-Adjusted Mortality Rates per 100,000, Stratified by States in the United States, 1999 to 2024

Supplemental Table 8: Cardiac arrest-related Age-Adjusted Mortality Rates per 100,000, Stratified by Census Region in the United States, 1999 to 2024

Supplemental Table 9: Cardiac arrest-related Age-Adjusted Mortality Rates per 100,000 in United States stratified by Urban-Rural Classification, 1999-2020

Supplemental Table 1: Overview of Variables Available in Death Certificate Data

Variable	Definition
**Year**	The calendar year in which deaths and population data were recorded.
**Deaths**	The total number of deaths attributed to the condition of interest within the specified population and year.
**Population**	The total number of individuals at risk in the specified population during the given year.
**Crude Rate per 100,000 (95% CI)**	The unadjusted mortality rate calculated as the number of deaths divided by the total population, expressed per 100,000 individuals, along with its 95% confidence interval indicating statistical precision.
**Crude Rate Standard Error**	A measure of the variability or uncertainty associated with the crude mortality rate estimate.
**Age-Adjusted Rate per 100,000 (95% CI)**	A mortality rate standardized to a reference population (e.g., standard population) to allow comparison across populations with different age structures, presented with a 95% confidence interval.
**Age-Adjusted Rate Standard Error**	The standard error associated with the age-adjusted mortality rate, reflecting the variability of the estimate.
**% of Total Deaths**	The proportion of deaths due to the condition of interest relative to all recorded deaths in the population, expressed as a percentage.

Supplemental Table 2: Cardiac arrest-related deaths, Stratified by Sex and Race in the United States, 1999 to 2024

Year	Overall	Female	Male	NH Black or African American	White	Hispanic or Latino	NH Others
1999	11767	4846	6921	3450	6430	1420	392
2000	10233	4284	5949	2718	5862	1184	415
2001	10218	4395	5823	2652	5863	1210	444
2002	10216	4312	5904	2617	5927	1215	414
2003	10169	4242	5927	2666	5690	1308	462
2004	10782	4386	6396	3075	5812	1458	397
2005	10840	4431	6409	3063	5722	1539	487
2006	10699	4405	6294	3052	5637	1485	501
2007	10275	4258	6017	2927	5419	1429	480
2008	10052	4207	5845	2850	5136	1534	514
2009	10114	4211	5903	2672	5369	1583	466
2010	9601	3990	5611	2547	5087	1473	466
2011	9469	3958	5511	2491	5128	1368	460
2012	9515	3966	5549	2409	5073	1500	506
2013	9852	3989	5863	2609	5150	1546	519

2014	10286	4293	5993	2664	5380	1682	529
2015	10383	4241	6142	2714	5317	1733	575
2016	10704	4334	6370	2734	5624	1707	607
2017	10859	4443	6416	2856	5555	1821	589
2018	11066	4608	6458	3004	5563	1854	624
2019	10957	4508	6449	2988	5365	1924	652
2020	14596	5793	8803	3908	6658	3032	950
2021	17430	7183	10752	4452	8021	3668	950
2022	14434	5927	9068	3864	6811	2772	737
2023	12748	5171	7577	3337	5960	2510	736
2024	11972	4712	7260	3165	5478	2401	714

NH, non-Hispanic.

Supplemental Table 3: Annual percent change (APC) of Cardiac arrest-related age-adjusted mortality rates per 100,000 in the United States, 1999 to 2024

Year Interval	APC (95% confidence interval)
Overall	
1999-2018	-0.05 (-0.62 to 0.40)
2018-2021	16.92* (10.80 to 20.41)
2021-2024	-14.36* (-21.10 to -7.40)
Female	
1999-2012	-0.61 (-4.94 to 0.33)
2012-2024	1.91* (0.82 to 6.96)
Male	
1999-2012	-0.51 (-6.44 to 0.62)
2012-2024	2.31* (1.06 to 8.46)
Black or African American	
1999-2018	-0.76 (-2.17 to 2.17)
2018-2021	17.17 (-7.75 to 22.76)
2021-2024	-13.45 (-25.80 to 5.64)
White	

1999-2018	0.48* (0.04 to 0.82)
2018-2021	13.01* (8.10 to 15.77)
2021-2024	-11.97* (-17.32 to -6.44)
Hispanic or Latino	
1999-2018	-0.88* (-1.71 to -0.19)
2018-2021	27.56* (18.62 to 32.97)
2021-2024	-19.41* (-26.80 to -11.77)
NH others	
1999-2017	-0.70 (-1.82 to 0.05)
2017-2020	15.18* (5.18 to 20.02)
2020-2024	-19.37* (-30.12 to -12.11)
Northeast	
1999-2013	-2.52* (-8.81 to -1.33)
2013-2024	1.04 (-0.99 to 11.82)
Midwest	
1999-2009	-1.03 (-3.04 to 0.48)
2009-2020	5.49* (4.38 to 10.61)
2020-2024	-3.53 (-11.03 to 2.19)
South	

1999-2023	0.52* (0.11 to 0.97)
West	
1999-2017	0.35 (-0.84 to 4.18)
2017-2020	9.70 (-3.46 to 13.06)
2020-2024	-2.62 (-10.46 to 3.99)
Urban	
1999-2018	-0.24 (-1.29 to 0.31)
2018-2020	16.06* (2.60 to 22.51)
Rural	
1999-2018	1.08* (0.44 to 1.48)
2018-2020	12.79* (3.38 to 17.42)

***Indicates that APC is significant (p<0.05).**

Supplemental Table 4: Trends in Cardiac Arrest related mortality among young adults in the United States Stratified by Location of Death, 1999 to 2024

Year	Medical Facility	Decedent's Home	Nursing Home	Hospice Facility
**1999**	9087	1785	441	Missing
**2000**	7886	1602	383	Missing
**2001**	7773	1662	417	Missing
**2002**	7739	1654	371	Missing
**2003**	7680	1700	331	39
**2004**	8026	1802	409	66
**2005**	8071	1763	430	87
**2006**	7955	1775	398	111
**2007**	7718	1661	352	132
**2008**	7175	1546	335	124
**2009**	7059	1554	309	146
**2010**	7177	1625	274	174
**2011**	7022	1652	274	161
**2012**	7082	1573	318	183
**2013**	7278	1707	315	160
**2014**	7553	1871	280	199
**2015**	7664	1831	297	191
**2016**	7889	1926	277	177
**2017**	8037	1926	286	192
**2018**	8268	1914	279	175
**2019**	8128	1958	231	193
**2020**	10990	2588	300	165
**2021**	17430	2809	364	167

**2022**	14434	2584	309	166
**2023**	12754	2206	292	171
**2024**	13492	2298	287	187
**Total**	231,367	48,972	8,559	3,366

Supplemental Table 5: Overall and Sex-Stratified Cardiac arrest-related Age-Adjusted Mortality Rates per 100,000 in the United States, 1999 to 2024

	Age-Adjusted Rate (95% confidence interval)		
Year	**Overall**	**Female**	**Male**
1999	9.58 (9.41 - 9.76)	7.91 (7.69 - 8.13)	11.25 (10.99 - 11.52)
2000	8.31 (8.15 - 8.47)	6.98 (6.77 - 7.19)	9.68 (9.43 - 9.93)
2001	8.28 (8.12 - 8.45)	7.17 (6.96 - 7.38)	9.47 (9.23 - 9.71)
2002	8.36 (8.2 - 8.52)	7.07 (6.86 - 7.28)	9.65 (9.4 - 9.89)
2003	8.38 (8.21 - 8.54)	6.99 (6.78 - 7.2)	9.77 (9.52 - 10.02)
2004	8.93 (8.76 - 9.1)	7.3 (7.08 - 7.52)	10.56 (10.3 - 10.82)
2005	9.02 (8.85 - 9.19)	7.37 (7.16 - 7.59)	10.63 (10.37 - 10.89)
2006	8.91 (8.74 - 9.07)	7.36 (7.14 - 7.58)	10.45 (10.19 - 10.71)
2007	8.58 (8.42 - 8.75)	7.11 (6.9 - 7.33)	10.06 (9.8 - 10.31)
2008	8.48 (8.31 - 8.64)	7.09 (6.87 - 7.3)	9.8 (9.54 - 10.05)
2009	8.58 (8.41 - 8.75)	7.15 (6.93 - 7.36)	9.99 (9.73 - 10.24)
2010	8.14 (7.98 - 8.31)	6.79 (6.58 - 7)	9.46 (9.21 - 9.71)
2011	8.05 (7.88 - 8.21)	6.8 (6.58 - 7.01)	9.37 (9.12 - 9.62)
2012	8.08 (7.91 - 8.24)	6.75 (6.54 - 6.96)	9.4 (9.15 - 9.65)
2013	8.37 (8.2 - 8.54)	6.82 (6.61 - 7.03)	9.93 (9.67 - 10.18)

2014	8.69 (8.52 - 8.86)	7.29 (7.07 - 7.51)	10.12 (9.86 - 10.37)
2015	8.71 (8.54 - 8.88)	7.18 (6.96 - 7.39)	10.28 (10.02 - 10.54)
2016	9 (8.83 - 9.17)	7.33 (7.11 - 7.55)	10.66 (10.4 - 10.92)
2017	9.05 (8.88 - 9.22)	7.45 (7.23 - 7.67)	10.6 (10.33 - 10.86)
2018	9.13 (8.96 - 9.31)	7.67 (7.44 - 7.89)	10.6 (10.34 - 10.86)
2019	8.96 (8.79 - 9.13)	7.42 (7.2 - 7.64)	10.5 (10.24 - 10.75)
2020	11.92 (11.72 - 12.11)	9.48 (9.23 - 9.73)	14.29 (13.99 - 14.59)
2021	13.94 (13.73 - 14.14)	8.72 (8.52 - 8.93)	12.78 (12.54 - 13.02)
2022	11.49 (11.31 - 11.68)	7.21 (7.03 - 7.4)	10.63 (10.41 - 10.85)
2023	10.05 (9.87–10.22)	8.19 (7.96–8.42)	11.84 (11.58–12.11)
2024	9.24 (9.08–9.41)	7.33 (7.12–7.54)	11.06 (10.81–11.32)

Supplemental Table 6: Cardiac arrest- related Age-Adjusted Mortality Rates per 100,000, Stratified by Race in the United States, 1999 to 2024

	Age-Adjusted Rate (95% confidence interval)			
Year	**Black or African American**	**White**	**Hispanic or Latino**	**NH others**
1999	22.39 (21.64 -23.14)	7.52 (7.34 -7.7)	9.43 (8.93 -9.93)	6.24 (5.62 -6.86)
2000	17.49 (16.83 -18.15)	6.9 (6.72 -7.08)	7.55 (7.11 -7.99)	6.34 (5.73 -6.95)
2001	17.01 (16.36 -17.66)	7.04 (6.86 -7.22)	7.31 (6.9 -7.73)	6.57 (5.95 -7.18)
2002	16.8 (16.16 -17.45)	7.19 (7.01 -7.37)	7.07 (6.66 -7.47)	5.93 (5.36 -6.51)
2003	17.18 (16.52 -17.83)	7.07 (6.89 -7.26)	7.26 (6.86 -7.66)	6.46 (5.87 -7.05)
2004	19.9 (19.2 -20.61)	7.33 (7.14 -7.52)	7.96 (7.54 -8.37)	5.39 (4.85 -5.92)
2005	19.81 (19.11 -20.52)	7.33 (7.14 -7.52)	8.07 (7.66 -8.47)	6.41 (5.84 -6.98)
2006	19.75 (19.05 -20.45)	7.31 (7.12 -7.51)	7.49 (7.1 -7.88)	6.37 (5.81 -6.93)
2007	18.87 (18.18 -19.55)	7.17 (6.98 -7.36)	7.02 (6.65 -7.39)	5.99 (5.45 -6.52)
2008	18.37 (17.69 -19.04)	6.92 (6.73 -7.11)	7.29 (6.92 -7.66)	6.3 (5.75 -6.85)
2009	17.34 (16.67 -18)	7.39 (7.19 -7.59)	7.24 (6.88 -7.6)	5.62 (5.11 -6.13)
2010	16.51 (15.86 -17.16)	7.05 (6.85 -7.24)	6.66 (6.32 -7.01)	5.54 (5.04 -6.05)
2011	16.18 (15.54 -16.82)	7.27 (7.07 -7.47)	6.03 (5.71 -6.36)	5.34 (4.85 -5.83)

2012	15.56 (14.94 -16.19)	7.27 (7.07 -7.47)	6.52 (6.18 -6.85)	5.69 (5.19 -6.19)
2013	16.7 (16.06 -17.35)	7.39 (7.19 -7.6)	6.65 (6.31 -6.98)	5.73 (5.23 -6.22)
2014	16.97 (16.32 -17.62)	7.8 (7.59 -8.01)	7.05 (6.71 -7.39)	5.65 (5.17 -6.14)
2015	17.06 (16.41 -17.71)	7.68 (7.47 -7.89)	7.07 (6.74 -7.41)	5.96 (5.47 -6.45)
2016	16.97 (16.33 -17.62)	8.19 (7.97 -8.41)	6.95 (6.62 -7.28)	6.18 (5.69 -6.68)
2017	17.55 (16.9 -18.21)	8.08 (7.86 -8.29)	7.2 (6.87 -7.54)	5.81 (5.34 -6.28)
2018	18.33 (17.67 -19)	8.08 (7.86 -8.29)	7.3 (6.97 -7.64)	6.03 (5.55 -6.5)
2019	17.97 (17.32 -18.62)	7.8 (7.59 -8.01)	7.41 (7.07 -7.74)	6.24 (5.75 -6.72)
2020	23.21 (22.47 -23.94)	9.55 (9.32 -9.79)	11.78 (11.35 -12.2)	8.98 (8.4 -9.55)
2021	26.85 (26.05 -27.65)	11.59 (11.34 -11.85)	13.92 (13.46 -14.37)	7.28 (6.82 -7.75)
2022	23.13 (22.4 -23.87)	9.8 (9.57 -10.04)	10.35 (9.96 -10.74)	5.55 (5.15 -5.96)
2023	19.70 (19.03–20.39)	8.59 (8.37–8.81)	9.10 (8.75–9.47)	6.98 (6.48–7.51)
2024	18.18 (17.55 -18.84)	7.88 (7.67 -8.09)	8.4 (8.06 -8.74)	6.28 (5.83 -6.76)

Supplemental Table 7: Cardiac arrest-related Age-Adjusted Mortality Rates per 100,000, Stratified by States in the United States, 1999 to 2024

State	Age-Adjusted Rate (95% confidence interval)		
	**1999-2020**	**2021-2023**	**2024**
Alabama	23.01 (22.53 - 23.48)	40.88 (38.78 -42.98)	32.28 (29.64 -34.92)
Alaska	5.24 (4.67 - 5.8)	14.06 (11.21 -17.4)	18.54 (13.97 -24.13)
Arizona	7.9 (7.66 - 8.14)	14.16 (13.15 -15.17)	9.99 (8.79 -11.19)
Arkansas	13.55 (13.08 - 14.02)	25.92 (23.78 -28.06)	20.42 (17.73 -23.12)
California	11.21 (11.09 - 11.32)	17.32 (16.86 -17.79)	14.5 (13.9 -15.1)
Colorado	5.07 (4.87 - 5.28)	8.27 (7.46 -9.08)	5.53 (4.59 -6.46)
Connecticut	9.67 (9.31 - 10.02)	7.79 (6.71 -8.88)	4.15 (3.13 -5.41)
Delaware	4.96 (4.45 - 5.46)	9.37 (7.23 -11.95)	7.68 (5.1 -11.09)
District of Columbia	5.28 (4.69 - 5.87)	4.3 (2.81 -6.3)	Unreliable (1.82 - 6.15)
Florida	9.6 (9.45 - 9.76)	13.03 (12.47 -13.6)	9.77 (9.07 -10.46)
Georgia	17.36 (17.08 - 17.63)	18.96 (18.02 -19.9)	14.5 (13.34 -15.66)
Hawaii	11.06 (10.44 - 11.67)	9.38 (7.55 -11.22)	10.07 (7.56 -13.13)
Idaho	6.99 (6.53 - 7.45)	8.75 (7.22 -10.28)	8.1 (6.17 -10.45)
Illinois	4.09 (3.97 - 4.21)	7.6 (7.04 -8.15)	6.89 (6.14 -7.64)

Indiana	7.05 (6.83 - 7.28)	10.83 (9.91 -11.75)	7.44 (6.36 -8.52)
Iowa	5.38 (5.09 - 5.67)	9.19 (7.94 -10.43)	7.98 (6.43 -9.79)
Kansas	6.28 (5.96 - 6.61)	10.29 (8.93 -11.65)	7.61 (6.06 -9.45)
Kentucky	9.16 (8.85 - 9.47)	15.89 (14.51 -17.27)	12.9 (11.13 -14.66)
Louisiana	9.16 (8.86 - 9.46)	17.01 (15.63 -18.39)	13.47 (11.73 -15.21)
Maine	3.73 (3.36 - 4.09)	2.24 (1.39 -3.42)	Unreliable (1.17 -4.2)
Maryland	2.69 (2.55 - 2.83)	6.16 (5.45 -6.87)	6.77 (5.71 -7.83)
Massachusetts	7.13 (6.91 - 7.34)	9.33 (8.5 -10.16)	9.17 (8 -10.34)
Michigan	4.31 (4.17 - 4.45)	8.23 (7.55 -8.9)	7 (6.12 -7.87)
Minnesota	3.23 (3.07 - 3.4)	6.9 (6.12 -7.68)	6.74 (5.65 -7.84)
Mississippi	30.73 (30.04 - 31.43)	48.39 (45.42 -51.36)	43.28 (39.32 -47.24)
Missouri	6.91 (6.68 - 7.15)	11.52 (10.53 -12.51)	9.51 (8.23 -10.79)
Montana	5.92 (5.38 - 6.46)	11.4 (9.2 -13.96)	9.54 (6.81 -12.99)
Nebraska	9.42 (8.93 - 9.92)	11.92 (10.15 -13.7)	10.88 (8.62 -13.54)
Nevada	15.44 (14.93 - 15.95)	24.84 (22.87 -26.81)	21.75 (19.15 -24.36)
New Hampshire	3.81 (3.44 - 4.17)	7.84 (6.19 -9.8)	8.29 (5.92 -11.29)
New Jersey	7.47 (7.28 - 7.66)	14.61 (13.7 -15.52)	11.52 (10.38 -12.65)
New Mexico	5.83 (5.46 - 6.2)	14.96 (13.02 -16.89)	10.3 (8.16 -12.81)
New York	15.66 (15.47 - 15.85)	13.3 (12.71 -13.9)	12.05 (11.25 -12.84)

North Carolina	7.49 (7.3 - 7.67)	13.09 (12.28 -13.9)	6.81 (5.98 -7.63)
North Dakota	7.82 (7.09 - 8.56)	17.82 (14.44 -21.2)	10.65 (7.24 -15.12)
Ohio	8.28 (8.09 - 8.46)	13.17 (12.39 -13.95)	10.79 (9.79 -11.78)
Oklahoma	7.13 (6.83 - 7.43)	6.87 (5.93 -7.81)	4.82 (3.79 -6.05)
Oregon	3.19 (3 - 3.38)	6.72 (5.83 -7.6)	6.77 (5.52 -8.02)
Pennsylvania	6.92 (6.76 - 7.08)	9.66 (9.03 -10.3)	7.44 (6.66 -8.23)
Rhode Island	6.77 (6.23 - 7.31)	5.96 (4.4 -7.91)	5.49 (3.44 -8.3)
South Carolina	12.59 (12.24 - 12.95)	15.28 (14.02 -16.55)	8.44 (7.12 -9.75)
South Dakota	7.07 (6.42 - 7.71)	17.53 (14.31 -20.75)	6.49 (4.02 -9.92)
Tennessee	11.11 (10.83 - 11.39)	15.65 (14.56 -16.74)	12.56 (11.19 -13.94)
Texas	5.91 (5.81 - 6.01)	9.11 (8.72 -9.49)	6.22 (5.78 -6.67)
Utah	4.57 (4.3 - 4.84)	5.74 (4.83 -6.65)	4.65 (3.56 -5.96)
Vermont	3.29 (2.79 - 3.79)	5.06 (3.21 -7.6)	Unreliable (4.37 - 12.02)
Virginia	6.01 (5.83 - 6.19)	6.9 (6.27 -7.54)	5.83 (5.01 -6.66)
Washington	4.69 (4.51 - 4.86)	7.92 (7.22 -8.62)	7.49 (6.53 -8.44)
West Virginia	10.69 (10.16 - 11.22)	10.13 (8.34 -11.92)	4.22 (2.75 -6.18)
Wisconsin	3.68 (3.51 - 3.86)	6.07 (5.32 -6.82)	5.56 (4.55 -6.57)

Wyoming	9.06 (8.18 - 9.94)	10.2 (7.41 -13.7)	Unreliable (4.45 - 12.24)

Supplemental Table 8: Cardiac arrest-related Age-Adjusted Mortality Rates per 100,000, Stratified by Census Region in the United States, 1999 to 2024

Census region	Year	Age-Adjusted Rate (95% confidence interval)
Northeast		
Northeast	1999	13.79 (13.31 - 14.26)
Northeast	2000	11.02 (10.6 - 11.44)
Northeast	2001	10.57 (10.15 - 10.99)
Northeast	2002	10.68 (10.25 - 11.1)
Northeast	2003	10.59 (10.17 - 11.01)
Northeast	2004	12.04 (11.58 - 12.49)
Northeast	2005	11.56 (11.11 - 12)
Northeast	2006	10.85 (10.42 - 11.29)
Northeast	2007	10.78 (10.34 - 11.21)
Northeast	2008	10.01 (9.58 - 10.43)
Northeast	2009	9.84 (9.41 - 10.26)
Northeast	2010	8.76 (8.36 - 9.16)
Northeast	2011	8.91 (8.5 - 9.32)
Northeast	2012	8.68 (8.27 - 9.08)
Northeast	2013	8.68 (8.27 - 9.09)

Northeast	2014	8.77 (8.37 - 9.18)
Northeast	2015	8.49 (8.09 - 8.89)
Northeast	2016	8.89 (8.48 - 9.31)
Northeast	2017	8.86 (8.45 - 9.27)
Northeast	2018	8.7 (8.29 - 9.11)
Northeast	2019	8.2 (7.8 - 8.59)
Northeast	2020	12.58 (12.09 - 13.08)
Northeast	2021	9.38 (9.02 - 9.74)
Northeast	2022	8.31 (7.97 - 8.65)
Northeast	2023	9.52 (9.11 - 9.95)
Northeast	2024	9.05 (8.65 - 9.46)
Midwest		
Midwest	1999	5.2 (4.93 - 5.47)
Midwest	2000	4.85 (4.59 - 5.11)
Midwest	2001	5.2 (4.93 - 5.47)
Midwest	2002	5.05 (4.78 - 5.32)
Midwest	2003	4.85 (4.58 - 5.11)
Midwest	2004	4.83 (4.57 - 5.1)
Midwest	2005	4.71 (4.45 - 4.97)

Midwest	2006	4.69 (4.42 - 4.95)
Midwest	2007	4.47 (4.21 - 4.73)
Midwest	2008	4.77 (4.5 - 5.04)
Midwest	2009	4.82 (4.55 - 5.09)
Midwest	2010	5.05 (4.77 - 5.33)
Midwest	2011	5.33 (5.04 - 5.62)
Midwest	2012	5.18 (4.89 - 5.47)
Midwest	2013	5.74 (5.43 - 6.04)
Midwest	2014	6.11 (5.8 - 6.42)
Midwest	2015	6.2 (5.89 - 6.51)
Midwest	2016	6.56 (6.24 - 6.89)
Midwest	2017	6.55 (6.23 - 6.88)
Midwest	2018	6.95 (6.61 - 7.28)
Midwest	2019	7.73 (7.38 - 8.08)
Midwest	2020	9.51 (9.12 - 9.9)
Midwest	2021	7.91 (7.61 - 8.21)
Midwest	2022	7.11 (6.82 - 7.39)
Midwest	2023	7.91 (7.56 - 8.26)
Midwest	2024	7.15 (6.83 - 7.49)

South		
South	1999	10.62 (10.31 - 10.92)
South	2000	9.33 (9.04 - 9.62)
South	2001	9.07 (8.79 - 9.35)
South	2002	9.47 (9.18 - 9.76)
South	2003	9.24 (8.96 - 9.53)
South	2004	9.91 (9.61 - 10.2)
South	2005	10.17 (9.87 - 10.47)
South	2006	10.22 (9.92 - 10.52)
South	2007	9.87 (9.58 - 10.16)
South	2008	9.63 (9.34 - 9.92)
South	2009	9.8 (9.5 - 10.09)
South	2010	9.39 (9.1 - 9.68)
South	2011	8.99 (8.7 - 9.27)
South	2012	9.13 (8.85 - 9.42)
South	2013	9.45 (9.16 - 9.74)
South	2014	9.97 (9.67 - 10.26)
South	2015	9.85 (9.56 - 10.15)
South	2016	10.16 (9.87 - 10.46)

South	2017	10.28 (9.98 - 10.57)
South	2018	10.11 (9.82 - 10.4)
South	2019	9.6 (9.32 - 9.88)
South	2020	12.21 (11.9 - 12.53)
South	2021	12.17 (11.9 - 12.44)
South	2022	9.6 (9.36 - 9.84)
South	2023	10.33 (10.05 - 10.62)
South	2024	9.5 (9.23 - 9.77)
West		
West	1999	8.77 (8.42 - 9.12)
West	2000	7.94 (7.61 - 8.27)
West	2001	8.29 (7.96 - 8.63)
West	2002	7.96 (7.63 - 8.29)
West	2003	8.58 (8.23 - 8.92)
West	2004	8.7 (8.35 - 9.04)
West	2005	9.12 (8.77 - 9.48)
West	2006	9.14 (8.79 - 9.5)
West	2007	8.61 (8.27 - 8.96)
West	2008	8.73 (8.38 - 9.07)

West	2009	8.99 (8.63 - 9.34)
West	2010	8.42 (8.08 - 8.76)
West	2011	8.43 (8.09 - 8.77)
West	2012	8.47 (8.13 - 8.81)
West	2013	8.73 (8.39 - 9.08)
West	2014	8.79 (8.45 - 9.14)
West	2015	9.16 (8.81 - 9.5)
West	2016	9.28 (8.93 - 9.63)
West	2017	9.13 (8.79 - 9.48)
West	2018	9.71 (9.35 - 10.06)
West	2019	9.51 (9.16 - 9.86)
West	2020	12.8 (12.4 - 13.2)
West	2021	11.88 (11.55 - 12.21)
West	2022	9.87 (9.57 - 10.18)
West	2023	11.66 (11.29 - 12.05)
West	2024	10.47 (10.12 - 10.84)

Supplemental Table 9: Cardiac arrest-related Age-Adjusted Mortality Rates per 100,000 in United States stratified by Urban-Rural Classification, 1999-2020

	Age-Adjusted Rate (95% confidence interval)	
Year	**Urban**	**Rural**
1999	9.35 (9.17 - 9.54)	10.79 (10.32 - 11.27)
2000	8 (7.83 - 8.18)	10.11 (9.65 - 10.57)
2001	7.9 (7.73 - 8.08)	10.89 (10.41 - 11.38)
2002	7.94 (7.76 - 8.11)	10.94 (10.45 - 11.43)
2003	7.97 (7.79 - 8.14)	11.02 (10.53 - 11.52)
2004	8.55 (8.37 - 8.73)	11.16 (10.66 - 11.66)
2005	8.54 (8.36 - 8.72)	11.94 (11.42 - 12.46)
2006	8.45 (8.27 - 8.63)	11.73 (11.21 - 12.26)
2007	8.14 (7.97 - 8.31)	11.18 (10.67 - 11.69)
2008	7.99 (7.82 - 8.16)	11.42 (10.9 - 11.94)
2009	8.13 (7.95 - 8.3)	11.34 (10.82 - 11.86)
2010	7.65 (7.48 - 7.82)	11.29 (10.77 - 11.82)
2011	7.45 (7.28 - 7.62)	12.04 (11.5 - 12.59)
2012	7.51 (7.34 - 7.68)	11.98 (11.43 - 12.52)
2013	7.81 (7.64 - 7.98)	12 (11.46 - 12.55)

2014	8.12 (7.95 - 8.3)	12.7 (12.14 - 13.27)
2015	8.12 (7.94 - 8.29)	12.79 (12.22 - 13.36)
2016	8.29 (8.12 - 8.47)	13.76 (13.16 - 14.35)
2017	8.46 (8.28 - 8.63)	13.04 (12.46 - 13.61)
2018	8.64 (8.46 - 8.82)	12.7 (12.13 - 13.27)
2019	8.43 (8.26 - 8.61)	12.91 (12.34 - 13.49)
2020	11.11 (10.91 - 11.31)	17.25 (16.59 - 17.92)
